# Microbubble-enhanced ultrasound for the antivascular treatment and monitoring of hepatocellular carcinoma

**DOI:** 10.7150/ntno.39514

**Published:** 2019-10-01

**Authors:** Julia C. D'Souza, Laith R. Sultan, Stephen J. Hunt, Terence P. Gade, Mrigendra B. Karmacharya, Susan M. Schultz, Angela K. Brice, Andrew K. W. Wood, Chandra M. Sehgal

**Affiliations:** 1 Ultrasound Research Laboratory, Department of Radiology, Perelman School of Medicine, University of Pennsylvania, 3620 Hamilton Walk, Philadelphia, PA 19104, USA; 2 Penn Image-Guided Interventions Lab, Department of Radiology, Perelman School of Medicine, University of Pennsylvania, 421 Curie Blvd, 646 BRB II/III Philadelphia, PA 19104, USA; 3 University Laboratory Animal Resources, University of Pennsylvania, 3800 Spruce Street, Philadelphia, PA 19104, USA.; 4 Department of Clinical Studies, School of Veterinary Medicine, University of Pennsylvania, 3900 Delancey Street, Philadelphia, PA 19104, USA.

**Keywords:** Hepatocellular carcinoma, noninvasive cancer therapies, antivascular ultrasound, preclinical studies

## Abstract

**Background and Objective**: Hepatocellular carcinoma (HCC) is the most common primary liver malignancy, and its current management relies heavily on locoregional therapy for curative therapy, bridge to transplant, and palliative therapy. Locoregional therapies include ablation and hepatic artery therapies such as embolization and radioembolization. In this study we evaluate the feasibility of using novel antivascular ultrasound (AVUS) as a noninvasive locoregional therapy to reduce perfusion in HCC lesions in a rat model and, monitor the effect with contrast-enhanced ultrasound imaging.

**Methods:** HCC was induced in 36 Wistar rats by the ingestion of 0.01% diethylnitrosamine (DEN) for 12 weeks. Two therapy regimens of AVUS were evaluated. A primary regimen (n = 19) utilized 2-W/cm^2^, 3-MHz ultrasound (US) for 6 minutes insonation with 0.7 ml of microbubbles administered as an intravenous bolus. An alternate dose at half the primary intensity, sonication time, and contrast concentration was evaluated in 11 rats to assess the efficacy of a reduced dose. A control group (n = 6) received a sham therapy. Tumor perfusion was measured before and after AVUS with nonlinear contrast ultrasound (NLC) and power Doppler (PD). The quantitative perfusion measures included perfusion index (PI), peak enhancement (PE), time to peak (TTP), and perfusion area from NLC and PD scans. Total tumor area perfused during the scan was measured by a postprocessing algorithm called delta projection. Tumor histology was evaluated for signs of tissue injury and for vascular changes using CD31 immunohistochemistry.

**Results:** DEN exposure induced autochthonous hepatocellular carcinoma lesions in all rats. Across all groups prior to therapy, there were no significant differences in the nonlinear contrast observations of peak enhancement and perfusion index. In the control group, there were no significant differences in any of the parameters after sham treatment. After the primary AVUS regimen, there were significant changes in all parameters (p ≤ 0.05) indicating substantial decreases in tumor perfusion. Peak enhancement in nonlinear contrast scans showed a 37.9% ± 10.1% decrease in tumor perfusion. Following reduced-dose AVUS, there were no significant changes in perfusion parameters, although there was a trend for the nonlinear contrast observations of peak enhancement and perfusion index to increase.

**Conclusion:** This study translated low-intensity AVUS therapy into a realistic *in vivo* model of HCC and evaluated its effects on the tumor vasculature. The primary dose of AVUS tested resulted in significant vascular disruption and a corresponding reduction in tumor perfusion. A reduced dose of AVUS, on the other hand, was ineffective at disrupting perfusion but demonstrated the potential for enhancing tumor blood flow. Theranostic ultrasound, where acoustic energy and microbubbles are used to monitor the tumor neovasculature as well as disrupt the vasculature and treat lesions, could serve as a potent tool for delivering noninvasive, locoregional therapy for hepatocellular carcinoma.

## Introduction

Hepatocellular carcinoma (HCC) is the most common primary liver malignancy and the third leading cause of cancer-related death worldwide 1. In the United States, two-year survival times have been reported at < 50% 2, 3. At present, curative therapies for very early and early stage HCC include thermal ablation, resection, and liver transplantation 4, 5. However, tumors may be beyond criteria for these interventions, often resulting in management of intermediate, advanced, and terminal stage HCC patients with transarterial embolization, radioembolization, or systemic chemotherapy for palliative treatment 6. Hence, nonsurgical locoregional therapies including ablation, transarterial embolization, and radioembolization through interventional oncology play a central role in curative therapy, as a bridge to transplantation, and as palliative treatment of HCC 7-9. Here, we study the application of antivascular ultrasound as a locoregional therapy for HCC, which in addition to requiring a less extensive and less expensive infrastructure to employ, utilizes a theranostic microbubble platform and is noninvasive.

Antivascular ultrasound (AVUS) therapy is a technique that uses low-intensity, unfocused ultrasound in combination with intravenously administered microbubbles to disrupt the tumor neovasculature 10-15. Microbubbles, commonly used as ultrasound contrast agents, consist of a lipid, polymer, or protein shell and a gas core. At 1 to 10 µm in diameter, they are small enough to pass through the lumina of capillaries yet large enough to prevent extravasation from vessels, ensuring their intravascular location 16-18. In AVUS, microbubbles are administered intravenously, and then ultrasound is applied to the tumor, stimulating the circulating microbubbles in the tumor vasculature. This causes the bubbles to oscillate at their resonance frequency (stable cavitation) or collapse (inertial cavitation), generating heat, shear stress, and microstreaming around the bubbles 19, 20. The slow blood flow of the disorganized tumor vasculature exposes the tumor endothelium to each microbubble for a longer residence time compared to the surrounding healthy vasculature, where the microbubbles pass more quickly. The microbubbles' localized bioeffects damage the adjacent endothelial cells and destroy the tumor's vascular supply, leading to ischemia and necrosis of neoplastic cells 10, 13, 15, 21 Rather than affecting tumor cells directly, the aim of AVUS is to destroy the tumor vasculature. This is analogous to pharmacologic vascular disrupting agents (VDAs) such as combretastatin, which target the tumor vasculature to cause ischemia and resultant necrosis of the tumor cells, inhibiting tumor growth and leading to regression of the tumor 23.

The effect of AVUS on tumor perfusion may be assessed by contrast-enhanced ultrasound (CEUS). CEUS employs microbubbles as contrast agents with shorter ultrasound pulses. Two CEUS modes of interest are nonlinear contrast and power Doppler modes. Nonlinear contrast ultrasound detects blood perfusion by detecting the nonlinear harmonic and subharmonic frequencies created by circulating microbubbles. This provides a high contrast-to-tissue ratio of tissue perfusion because biological tissue does not emit subharmonic frequencies 24, 25. Additionally, this allows detection of perfusion at greater tissue depths due to reduced attenuation of the lower frequency subharmonics 26, 27. Power Doppler ultrasound detects the strength of the Doppler signal scattered by blood cells and microbubbles, and the signal is sensitive to low-velocity blood flow in small diameter vessels (capillaries) 28, 29.

This study evaluates the theranostic ability of ultrasound and microbubbles in AVUS to disrupt and image tumor perfusion when translated into a more complex and realistic rat model of HCC. Two levels of tumor insonation were evaluated. One dose was translated from the dose successfully used in previous murine cancer studies, taking into consideration the larger size and blood volume of rats versus mice. Another reduced dose was used to determine whether AVUS was still effective while minimizing the ultrasound exposure to subjects.

## Materials and Methods

### Animal model

The University Institutional Animal Care and Use Committee approved all animal protocols. Thirty-six Wistar rats (Charles River Laboratories, Wilmington, MA, USA) weighing between 300 and 400g were acquired and kept under controlled environmental conditions (25°C and a 12-h light/dark cycle). After one week of acclimation, the rats were initiated on 0.01% diethylnitrosamine (DEN, Sigma Aldrich, St. Louis, MO, USA) in their drinking water, which they ingested ad libitum for 12 weeks. DEN was replaced 1 to 2 times per week as necessary, and rats were weighed twice weekly. Normal drinking water was returned at the beginning of the 13th week. For the sonographic procedures, the rat was placed in a non-rebreathing gaseous anesthetic system [VetEquip Inc, Livermore, CA, USA] and general anesthesia was induced with 4% isoflurane (IsoSol; VEDCO Inc, St Joseph, MO). A nose cone was taped in place, anesthesia was maintained with 1% to 3% isoflurane and 1.5 to 2.0 L.min-1 oxygen, and a tail vein 26G catheter [Covidien, Dublin, Ireland] was inserted. Animals were positioned supine on a heated bed (VisualSonics Vevo imaging station, Fujifilm, Toronto, Canada), the abdomen was depilated, and continuous vital signs including body temperature, heart rate, and respiratory rate were recorded. At the completion of each study, the rats were euthanized by carbon dioxide asphyxiation, and necropsy was performed.

### Antivascular ultrasound therapy

The rats were monitored for the development of tumors using grayscale ultrasound (VisualSonics VevoLAZR, Fujifilm, Toronto, Canada) beginning 11 weeks into the DEN diet and weekly thereafter. During monitoring sessions, diagnostic scans were performed identifying and measuring visualized lesions. A single tumor most suitable for AVUS therapy was selected from the often multiple tumors developed in the liver based on the tumor location (ventral and accessible to ultrasound imaging and therapy), size (6- to 14- mm diameter), hyperenhancement with microbubble administration, and minimal cystic or hemorrhagic changes as identified in the ultrasound images. After selecting and clearly identifying the tumor, the diagnostic transducer was secured in an adjustable mount (VisualSonics Vevo imaging station, Fujifilm, Toronto, Canada) to maintain a fixed position over the tumor. The same mount was used to position the low-intensity ultrasound therapy probe (Dynatronics D150 Plus, Dynatronics Salt Lake City, UT) to ensure the therapy beam's accurate alignment with the imaged HCC.

Two AVUS therapy regimens were used. A primary dose was selected based on our previous studies in mice 10, 12, 30 taking into account the greater body weight and blood volume of the rats. This primary regimen (n = 19) utilized 2.0-W/cm2; 3-MHz for a total of 6 minutes insonation [3 x 2-min insonations each separated by 2 min]). 0.5 mL perflutren microbubbles (Definity, Lantheus, N. Billerica, MA, USA) were injected as a bolus via the tail vein catheter prior to the first insonation and 0.2 mL prior to the second insonation; fractionating the microbubble administration ensured that microbubbles remained in circulation throughout the therapy. In the reduced dose regimen (n = 11) 1.0-W/cm2, 3-MHz was applied for a total of 3 minutes insonation [3 x 1-min insonations each separated by 1 min]), 0.1 mL microbubbles were injected before each insonation. A control group (n = 6) received a sham therapy in which the microbubbles were replaced with 0.7mL normal saline, and the US machine was not switched on.

### Multimodal ultrasound evaluation of HCC perfusion

B-mode ultrasound images (VisualSonics, VevoLAZR, 21-MHz linear transducer, Fujifilm, Toronto, Canada) of each HCC were acquired prior to therapy to assess the tumor size and positioning; the imaging was repeated after AVUS. This same equipment was used to acquire contrast-enhanced ultrasound (CEUS) scans to assess tumor perfusion before and after AVUS; fixed imaging parameters were used (18 MHz; contrast gain = 41; 2D gain = 18; sensitivity = 3; standard line density; power = 4). An intravenous bolus injection of up to 0.1 mL microbubbles was given for each of the CEUS studies and power Doppler (PD) observations were recorded and analysed (16 MHz; frame rate = 4 to 6 fps; Doppler gain = 37 dB; 2D gain = 18 dB; sensitivity = 3; standard line density; high, power = 100%; persistence = medium).

Tumor perfusion was assessed on complete CEUS data sets. In the nonlinear contrast scans (NLC), analyses utilized imaging software (VevoLab, Fujifilm, VisualSonics, Toronto, Canada). The borders of each tumor, were manually outlined in the initial B-mode image to create a region of interest (ROI). The mean intensity of the ROI was fitted to a bolus perfusion model 27, and the following parameters were assessed: peak enhancement (PE - the difference between the maximum amplitude and baseline intensity, which is proportional to microbubble concentration and indicative of relative blood volume); perfusion index (PI - the area under curve divided by mean transit time of flow); and time to peak (TTP - time from contrast administration to peak enhancement).

The PD scans were analyzed using a previously described IDL-based platform for vascular analysis 31. Tumor vascularity was measured within the tumor ROI and the Doppler image's color scale was used to detect pixels with flow (colored pixels) and the strength of Doppler signal (color bar). These were used to calculate area-based measurements and color-weighted fractional area (CWFA), representing contrast flow through the region over time. This allowed dynamic assessment of the tumor area perfused as well as flow changes in the perfused vessels.

The total tumor area perfused during the scan was measured by a postprocessing subtraction algorithm, delta projection, applied to grayscale images to assess contrast changes 32. By tracking the running maximum intensity in each pixel following the injection of microbubbles and subtracting the sequence's baseline intensity, the echogenic increases related to the flow of contrast are detected; thus, there is a global assessment of the proportion of the tumor being perfused.

### Histopathologic studies

At necropsy, tissue from the treated HCC and the adjacent normal liver were harvested for histologic examination. The tissues were preserved in 10% neutral buffered formalin followed by 70% ethanol and embedded in paraffin for sectioning. The institutional veterinary comparative pathology core prepared H&E sections and CD31 immunohistochemistry (AB28364; Abcam, Cambridge, MA) identifying the vascular endothelium.10x-magnified images of the tumor were stitched together (ZEN software, Carl Zeiss Microscopy, Oberkochen, Germany) to produce images of the entire tumor. Areas of hemorrhage and necrosis were segmented and analyzed in ImageJ 33 and recorded as the percentage area of histologic change.

### Statistical analysis

One-tailed, paired Student's t-tests on the decrease in tumor perfusion after therapy, as demonstrated in previous murine studies, were used to compare pre- and post-AVUS perfusion parameters within each treatment group. Additionally, ANOVA was used to compare pre-AVUS parameter values between groups, ensuring the groups were equivalent. ANOVA was used to compare percent change of perfusion parameters between the three treatment arms. One-tailed Student's t-tests were used to evaluate histologic changes, assuming vessels would dilate or expand with therapy and that therapy would increase tumor necrosis and hemorrhage relative to primary or sham tumors as shown in previous studies. An alpha of 0.05 was considered statistically significant. Values are reported as mean ± SEM. Statistics were calculated using Stata version 15.0, StataCorp, College Station, TX.

## Results

### Animal model monitoring

The initial weight of animals on acquisition was 364.2 ± 33.5 g (range 347 to 379g). The rate of weight gain reduced with exposure to DEN, and rats reached a final weight of 447 ± 80 g at the time of therapy. All animals developed liver disease and multiple foci of hepatocellular carcinoma (HCC), seen on B-mode ultrasound imaging via ascites and echogenic changes, gross pathology by nodularity, hepatosplenomegaly and multifocal lesions, and by histologic analysis confirming HCC diagnosis. The treatments ranged from day 88 to 119 from the start of the DEN diet (week 13 to 17).

### Tumors in all treatment groups showed similar perfusion prior to therapy

Contrast-enhanced US studies showed equivalent vascular perfusion kinetics throughout all tumors prior to AVUS therapy. By nonlinear contrast US imaging, the mean peak enhancement before therapy among all rats was 4.88 ± 0.88 a.u. (SEM) with no difference between sham, primary-dose, and reduced-dose groups (ANOVA F_2,33_ = 0.17, p = 0.85). The tumors also had equivalent pre-therapy rates of perfusion, modeled by perfusion index (PI): 4.24 ± 0.81 a.u. among all 39 tumors (F_2,33_ = 0.14, p = 0.14).

### The primary AVUS dose shows reduced tumor perfusion after therapy, quantitated via NLC perfusion parameters

**Figure 1:**
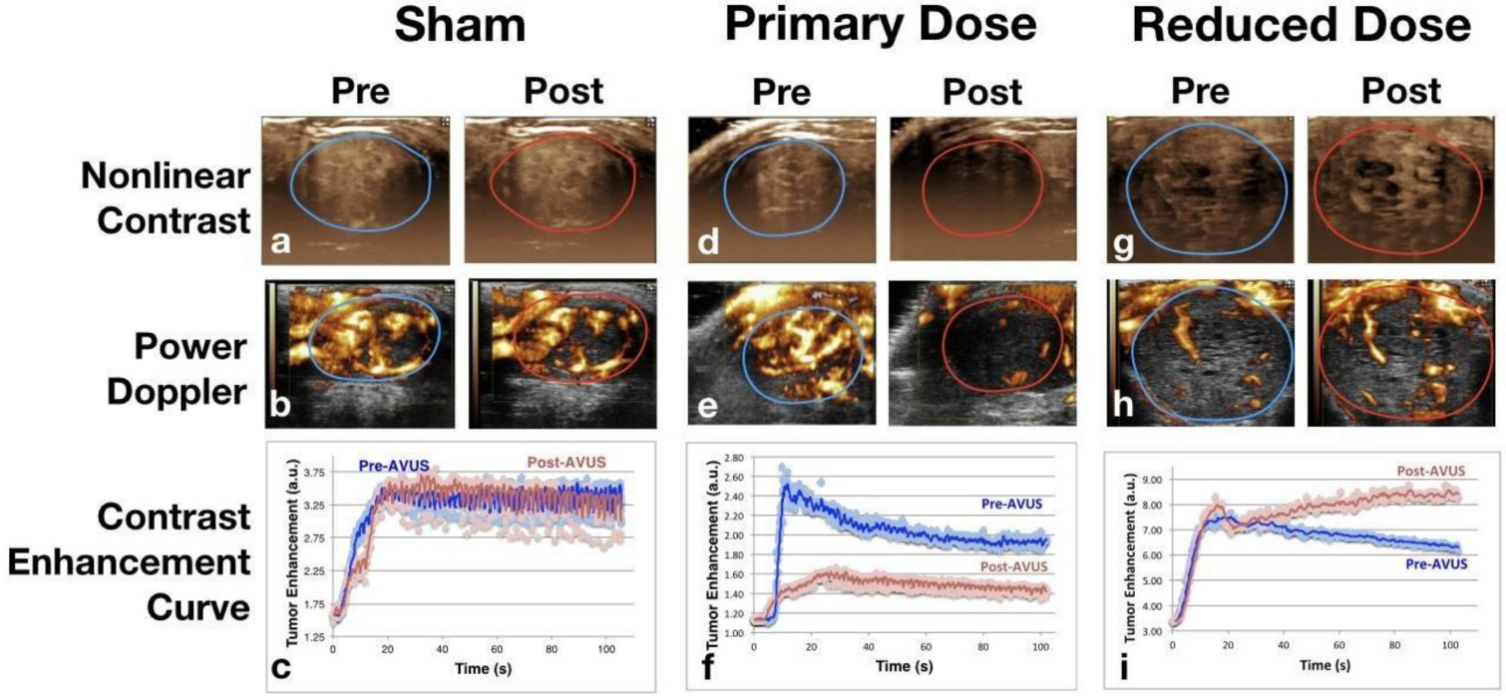
**Nonlinear Contrast and Power Doppler Images Pre- and Post-AVUS**. The top row of images shows maximum intensity projection images from nonlinear contrast scans with the tumor region of interest indicated. The sham treatments show equivalent perfusion before and after therapy. A notable decrease in tumor contrast enhancement can be noted in the Post image of therapy. Reduced-dose therapy, while showing a variable response overall, showed an increase in the majority (7 of 11 cases) that is shown above by the increase in contrast intensity. Power Doppler images demonstrate similar findings, with equivalent perfusion area and intensity before and after therapy in sham treatment, decrease in primary dose AVUS, and increase of Doppler signal in reduced dose. Contrast enhancement curves show examples of each therapy arm before and after treatment. Equivalent peak enhancement levels are seen in the sham treatment, while primary-dose peak enhancement and total contrast delivered decreases, and reduced-dose therapy shows a slightly higher peak enhancement with continuing contrast recirculation within the tumor.

Nonlinear contrast perfusion parameters showed a decrease in tumor perfusion after treatment with AVUS at the primary dose. An example can be appreciated visually in Figure 1 by the decreased enhancement of the post-treatment image of the primary dose (Figure 1d) versus an example of a sham treatment in Figure 1a, which shows equivalent pre and post enhancement. Additionally, the examples of pre and post time-intensity curves (Figure 1f) demonstrate the lower peak enhancement after therapy that quantitates this decrease.

**Figure 2:**
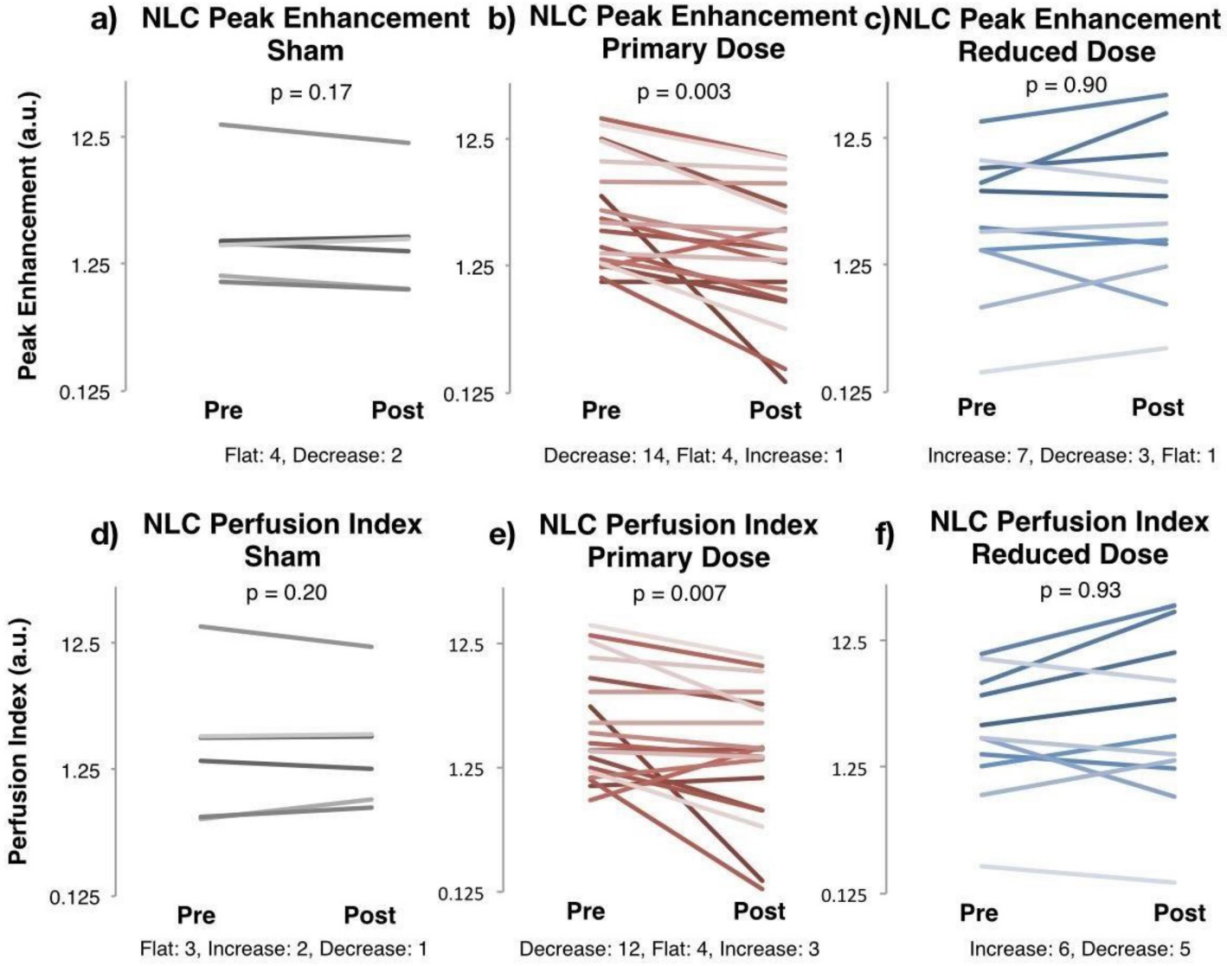
**Nonlinear Contrast Imaging Changes Pre- and Post- AVUS.** Nonlinear contrast (NLC) diagnostic ultrasound enhancement curves were fitted to a bolus perfusion model, and perfusion parameters including peak enhancement and perfusion index were used to evaluate changes in perfusion kinetics. Peak enhancement, representing blood volume perfusing the tumor, stayed constant before and after sham therapy, whereas it decreased in 14 of 19 cases, showing a change in mean of 5.30 ± 1.28 a.u. to 2.77 ± 0.65 a.u. in the primary dose arm (p = 0.003). Perfusion index, representing the rate of perfusion over time, also stayed constant in the sham arm but decreased in the primary dose arm. A reduced dose of AVUS showed an increase in the majority of cases by mean values, but was not found to be significant nor consistent.

This decrease in enhancement was evident in the primary dose arm. NLC peak enhancement, representing relative blood volume perfusing the tumor, decreased by >35% in 14 of 19 cases, as demonstrated in Figure 2b. Four rats showed no response, and one increased slightly. The average peak enhancement of the group fell from 5.30 ± 1.28 a.u. before therapy to 2.77 ± 0.65 a.u. after therapy (paired t-test p = 0.003). The average decrease between pre and post PE was 37.9% ± 10.05% within each tumor. In comparison, four of six sham-treated tumors showed no response to therapy (stayed flat with less than 10% change in Figure 2a). The group's average peak enhancement remained constant at 3.87 ± 2.41 a.u. before and 3.07 ± 1.64 a.u., after therapy (p = 0.17), decreasing only 8.8% ± 6.8% within each tumor (p = 0.02 versus primary dose).

The primary dose group's reduction in NLC peak enhancement was accompanied by a decrease in the rate of perfusion. The perfusion index (PI) decreased in 12 of the 19 rats (Figure 2e). The group's PI decreased from 4.65 ± 1.20 a.u. before therapy to 2.81 ± 0.65 a.u. after therapy (p = 0.007), and was accompanied by a lengthened time to peak enhancement (TTP), which slowed from 16.5 ± 1.8 seconds to 24.6 ± 1.8 seconds (p = 0.009). In comparison, the sham group showed no significant change in pre- and post-therapy PI or TTP (p = 0.20, p = 0.07) (Figure 2d). These parameters show reduction and slowing of tumor perfusion following primary dose AVUS.

### Power Doppler and delta projection also show reduced perfusion after the primary AVUS dose: reduced area of perfusion and a weaker intensity of contrast within perfused vessels is observed

Similarly, power Doppler showed a reduction in perfusion kinetics after primary AVUS dose. The peak perfused area (PPA) decreased from 87.2 ± 3.6 a.u. to 76.4 ± 9.1 a.u. as a group (p = 0.014). Assessing the change within this decreased area using the color weighting of the Doppler signal's strength (color-weighted flow area, CWFA), CWFA PE decreased from 48.0 ± 3.0 a.u. to 35.5 ± 3.6 a.u. (p = 0.007), indicating weaker flow within the smaller perfused area. The sham group remained constant for these parameters (SI Table S1).

Delta projection post-processing, measuring total tumor area receiving any perfusion throughout the scan, showed that less of the tumor area was perfused after therapy. Whereas 75.7% ± 4.2% area of primary-dose tumors enhanced before therapy, only 59.1 ± 6.4% enhanced after therapy (p = 0.002). Primary dose stayed constant at 57.6% ± 10.0% before and 58.2% ± 10.2% area perfused after therapy (p = 0.63).

### Reduced-dose AVUS showed increased perfusion but inconsistently

**Figure 3:**
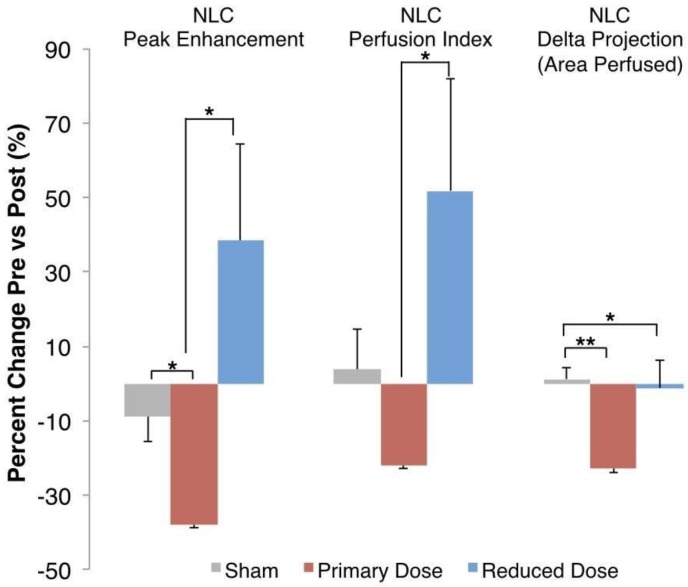
**Percent Change in Perfusion Parameters.** Nonlinear contrast ultrasound showed a decrease in blood volume perfused of 37.9% ± 10.05% in each tumor before and after primary-dose AVUS, while perfusion only decreased 8.8% ± 6.8% in the sham arm (p = 0.02). Perfusion per tumor in the reduced-dose arm increased compared to primary-dose AVUS (p = 0.02) but was not significant versus the sham arm (p = 0.11). Perfusion index showed a similar pattern, but did not show significant differences versus sham treatment. Delta projection, quantifying the tumor area perfused throughout the scan, showed a constant perfusion in sham (increase of 1.2% ± 3.2%) and a decreased area of the tumor receiving perfusion with contrast agent after primary-dose AVUS (decrease of 22.9% ± 7.3%, p = 0.005).

Though reduced-dose AVUS showed a net increase in perfusion as a group, this increase was variable between cases and was not statistically significant. NLC peak enhancement showed 7 cases increased, 3 cases decreased, and 1 case remained constant in perfusion (Figure 2c). As a group, peak enhancement rose from 4.71 ± 1.48 a.u. to 4.90 ± 2.72 a.u. (p = 0.90), with a 38.3% ± 26.2% average increase in perfusion in individual cases. Perfusion index did not change significantly, showing pre- and post-therapy values of 3.66 ± 1.00 a.u. and 6.65 ± 2.56 a.u., respectively (p = 0.92). Power Doppler and delta projection measures of perfused area showed no change from 67.5 ± 9.3 pre to and 68.2 ± 8.5 a.u. post therapy, respectively, for PAI (p = 0.23). Delta projection showed 66.8% ± 8.2% area perfused before and 64.3% ± 7.8% after therapy (p = 0.29). An example of these trends of increased strength of perfusion within a constant area can be noted in Figures 2g and 3h. However, this was found to be inconsistent and variably evoked.

The differences between the three arms tested are summarized in Figure 3, with the percent changes before and after therapy measured in individual tumors and then averaged for each group. While sham tumors demonstrate <10% change for each parameter, the primary AVUS dose consistently reduces perfusion parameters. The mean increases following reduced-dose AVUS indicate a domain of therapy that differs significantly from the primary-dose decreases in perfusion.

### Histologic analysis shows an increased number of medium and large vessels in tumors following AVUS

**Figure 4:**
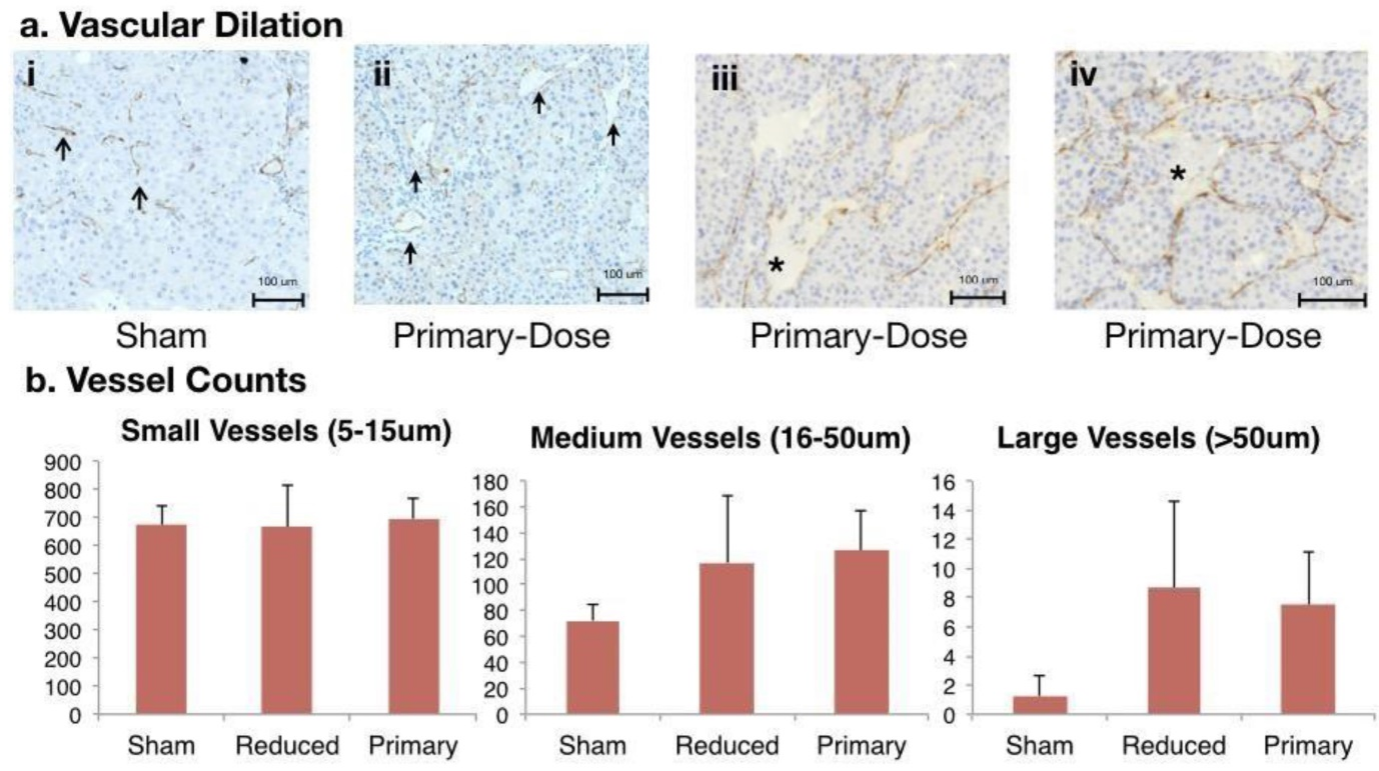
**CD31 Staining of Vascular Changes in AVUS-treated rats.** a) i. Small, narrow vessels comprising the majority of vessels in sham tumors. ii. A primary-dose tumor showing increased frequency of larger diameter vessels (solid arrows). iii. Pooling of RBCs (*) with endothelium on both sides, suggesting a wide dilation of a vessel. iv. Pooled RBCs (*) with hemorrhage on only one side, indicate possible rupture and extravasation. b) Counts of small, narrow diameter blood vessels (5 to 15um in diameter), medium vessels (16 to 50um), and large vessels (>50um) were measured throughout the tumor sections. While counts of small vessels were similar between all three treatment arms, a trend of increasing counts of medium (p = 0.06 primary-dose) and large vessels (p = 0.06 primary, p = 0.06 reduced) in treated tumors were noted, suggesting vascular dilation after therapy.

Endothelial cell staining using anti-CD31 immunohistochemistry suggested vessel dilation in primary-dose-treated tumors compared to sham tumors. Small, narrow-diameter blood vessels (5 to 15 μm in diameter) comprised the majority of vessels in the sham-treated tumors as well as in treated tumors. Primary-dose tumors showed pools of red blood cells (RBCs, * in Figure 4a) with endothelium on both sides, suggesting a wide dilation of vessels. Additionally, pooled RBCs with hemorrhage on only one side (Figure 4e) indicate rupture and extravasation of RBCs from vessels. Measuring and counting vessels allowed further understanding of these vascular changes. Counts of small vessels were equivalent between treatment arms with 684 ± 49 vessels/ six high-power fields. More medium (16 to 50 μm) and large (>50 μm) vessels were observed in tumors treated with primary-dose AVUS. On average there were 126 ± 32 medium vessels in the treated tumors, compared to 72 ± 12 medium vessels in the sham. Large vessels were also more frequent in treated tumors, with 7.5 ± 3.6 vessels counted compared to only 1.9 ± 1.3 vessels in sham tumors. These trends of increasing medium (p = 0.06 primary dose) and large vessels (p = 0.06 primary dose, p = 0.06 reduced dose) in treated tumors suggests vascular dilation after therapy.

**Figure 5:**
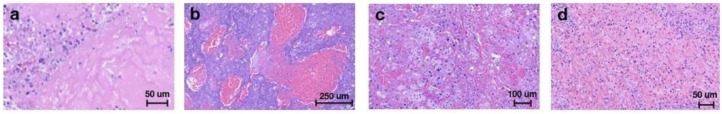
**Tissue damage over time included increased hemorrhage and necrosis on H&E.** Necrosis (a), pools of hemorrhage (b), and diffuse infiltrating hemorrhage (c) were observed after immediate sacrifice. Necrosis, hemorrhage, fibrin replacement, and neutrophilic infiltrate (d) were present in a small cohort sacrificed 1-4 days after AVUS.

Tumor sections stained with H&E staining were analyzed for the percent area of hemorrhage and necrosis to investigate damage immediately after AVUS. In the sham group, an average of 10% ± 2% area of the tumor section showed damage, which was statistically equal to the 8% ± 4% and 9% ± 2% of damage in reduced- and primary-dose-treated tumors sacrificed two hours after AVUS (ANOVA F_2,27_ = 2.00, p = 0.15). The range of damage included necrosis, pools of hemorrhage, and hemorrhage infiltrating between tumor cells (Figure 5).

Gross hemorrhage or necrosis was not observed in the adjacent liver. Changes in the adjacent liver included single cell necrosis and multiple foci of coagulative necrosis, consistent with ischemia occurring in cirrhosis, and was present in sham and AVUS-treated animals.

## Discussion

Antivascular ultrasound therapy is a novel localized therapy that applies low-intensity ultrasound to tumors in combination with intravenously-administered circulating microbubbles to induce vascular changes 10, 31, 12, 13, 15. This technique has been demonstrated to provide a noninvasive means of disrupting the tumor vasculature in a subcutaneous murine model of melanoma 21, 34, 13, 15, but has not yet been investigated as a therapy for HCC. The use of an autochthonous model of HCC induced by DEN hepatotoxin in this study allows for a realistic evaluation of HCC with authentic features in the setting of chronic liver disease and cirrhosis 35. This provides a necessary step in translation of treating neoplasms in situ with the proper vascular modeling for this vascular therapy.

In this realistically complex model the chemical hepatotoxin diethylnitrosamine (DEN) produces primary metabolic activation and DNA alkylation, leading to hepatic necrosis, inflammation, fibrosis, and eventual carcinogenesis that closely mimics chronic liver disease and development of HCC in humans 36, 37. Compared to the subcutaneous melanoma and colorectal cancer murine models previously studied, this provides a more realistic environment and recapitulates the unique dual blood supply of HCC lesions and the surrounding liver. This model also allows for factors that would be encountered in treating HCC in humans, such as ultrasound attenuation, heat dissipation to surrounding tissues and blood vessels, and damage to the surrounding liver and organs.

The primary dose of AVUS studied was able to reduce perfusing blood volume in the tumor and rate of blood flow. On nonlinear contrast ultrasound (NLC) studies immediately after therapy, the primary dose showed a reduction in peak enhancement, which represents relative blood volume perfusing the tumor, with an individual decrease of about 38% ± 10% in each tumor. Additionally, blood flow was slower after therapy. Time to peak and perfusion index both slowed. The decreased rate and intensity of flow observed on NLC were confirmed by power Doppler measures, including CWFA peak enhancement, which revealed that within the reduced area being perfused, the volume of blood and contrast being delivered is decreased 38, 39. These independent CEUS investigations of blood flow with high contrast-to-tissue ratio support the ability of AVUS to disrupt tumor perfusion, in accordance with previous studies. It is also close to the 45% ± 6% decrease in perfused area previously observed when treating subcutaneous murine melanoma with 1 minute of AVUS 10. In the prior study, area perfused was further decreased by 67% ± 8% with a 3-minute treatment of AVUS at the same parameters, indicating the potential for a greater effect of AVUS by increasing the time of treatment.

The variability in the treatment response shown in Figure 2 could be a result of multiple factors related to tumor biology and experimental protocol. Differences in tumor hemodynamics between animals could cause variability in the treatment response. For example, tumors with faster flow are likely to have weaker response to the treatment because of the reduced interaction between microbubbles and ultrasound due to rapid transit of the microbubbles through the ultrasound beam. In our studies, a fixed dose of contrast was used in all cases. Variability in animal weight and tumor size between individual cases was not accounted for and could have contributed to differences in the response. Finally, imaging was performed in a fixed 2D plane which is unlikely to capture antivascular response in all parts of the tumor and may cause variability in the measured treatment response. The recent advances in 3D imaging 40 could potentially overcome the current limitations of 2D imaging.

Considering the perfusion changes together with histological findings of vascular enlargement supports an effect on the tumor vasculature ranging from dilation to hemorrhage. The early sacrifice timepoint in this study allowed a closer understanding of the immediate mechanism of AVUS as opposed to the subsequent bioeffects of ischemia, hypoxia, and necrosis that have been previously noted 10, 21. Similar to the observations of Bunte et al 21 showing more prominent vessels and dilation on H&E, the CD31 immunohistochemistry in this study allowed measurement of vascular diameter and identification of dilated and ruptured blood vessels and pools of hemorrhage that were not enclosed by endothelium. The increased incidence of medium- and large-diameter vessels in tumors treated with primary-dose AVUS points toward dilation and impaired integrity or rupture that can lead to hemorrhage.

These findings support the efficacy of AVUS as a physical antivascular therapy for HCC in the context of its authentic anatomical location, microenvironment, and blood supply. The proven perfusional effect of AVUS after translating from a subcutaneous model to an autochthonous model addresses concerns about ultrasound attenuation in treating tumors within visceral organs versus subcutaneous tumors and takes into account the authentic tumor vasculature. The spatial confinement of the ultrasound beam to the tumor without targeting surrounding liver, which should have preserved perfusion, was also assessed. In this study, deleterious effects beyond those due to cirrhosis were not observed in the surrounding liver. However, the timepoint investigated does not preclude the development of necrosis over time.

Unexpectedly, a nonlinear dose effect was seen when evaluating a reduced dose of AVUS. Contrary to the milder disruptive effect that was expected, the reduced dose showed a variable effect, with enhancement of flow in the majority of cases. The average increase of 38% ± 26% may be attributed to tissue heating induced at the lower intensity of ultrasound and decreased concentration of microbubbles. The use of low-intensity ultrasound in the absence of microbubbles as in physical therapy has been demonstrated to cause increases in muscle perfusion as early as 1955, in a study demonstrating an increase in blood flow after insonation with 1-W/cm^2^ US for 15 minutes 41. Thus, with the reduced intensity, it is possible that hyperthermic effects involving increased flow dominates in this regimen compared to the higher intensity's predominantly disruptive effects associated with stronger thermal and mechanical forces on the vascular endothelium.

The effects of AVUS observed in this study are influenced by both the concentration of microbubbles and ultrasound parameters. An increased microbubble concentration provides more sonosensitizers to affect the vasculature. Additionally, with higher concentrations, microbubbles are more tightly packed, placing more bubbles in closer proximity to the tumor's endothelial cells. Previous studies have shown that the effects of microbubbles extend immediately around the microbubbles. Therefore, better approximation of the bubbles to the endothelium could enhance the therapeutic effect. Secondly, the intensity of US used causes greater amplitudes of bubble oscillation, generating higher shear and heating by heat viscous damping of the bubble oscillations.

In addition to antivascular ultrasound targeting tumor microvasculature, contrast-enhanced ultrasound enables imaging of tumor vasculature with high sensitivity and specificity. This ultrasound-microbubble combination provides a unique theranostic platform where externally triggered treatment and monitoring can be performed serially by simply changing the mode of ultrasound operation. The use of remotely triggered theranostics allows for early recognition of the disease and the controlled, image-guided treatment 42. Although the focus of this study was to use a predetermined AVUS treatment, it is reasonable to anticipate contrast-enhanced imaging to guide the treatment, where the dose is adjusted based on the changes in tumor vasculature observed following treatment. The recent approval of microbubble contrast agent for hepatic imaging in the US and the well-established role of microbubbles in clinical imaging in many countries around the world furthers the potential of translation of the study to human subjects.

Additionally, AVUS's use of microbubbles that can be customized provides a platform for further development to improve localized drug delivery, tumor targeting, and imaging. It is well known that the leaky tumor vasculature is a limiting factor for the therapeutic response via the extravasation of drugs from hypopermeable tumor vessels. The use of AVUS can safely and possibly reversibly increase the permeability of the tumor vessel walls and temporarily enhance the accumulation of anticancer drugs 43. Furthermore, the microbubbles may be loaded with drug cargo or conjugated with drug nanoparticles or liposomes. Encapsulating cargo that is unstable or easily degraded in the plasma could protect antibodies, RNA, or DNA 44. Bursting loaded MBs within the tumor would allow local release while sparing tissues outside the US beam, and could increase the permeability and uptake of the local tumor vasculature where the drug is released. The microbubbles also may be conjugated to ligands that target tumors. Microbubbles targeted to integrins commonly found on tumor neovasculature have already been developed for molecular imaging of tumors 45 and may be used to enhance selective delivery of therapy to tumors. Lastly, microbubbles are useful for monitoring the response to anticancer therapies affecting tumor perfusion as they enhance within the tumor microvasculature 23, 30. Aside from being ultrasound contrast agents, conjugation of nanoparticles or liposomes with super-paramagnetic iron oxide (SPIO) particles or gadolinium conjugates could also enhance imaging sensitivity by other modalities such as magnetic resonance imaging.

Limitations to this study and concerns for AVUS exist and should be further investigated. First, this study focuses on the immediate effects of AVUS on HCC, within the first two hours of therapy. While this immediate effect is useful in understanding the effect AVUS has on the tumor vasculature and perfusion, further studies of the bioeffects over time will inform whether the therapy contributes to a response and regression of HCC. This should look at delayed timepoints allowing for necrosis and other biological responses including the previously noted immune response 10 to manifest and ideally include survival studies. Additionally, surrounding organs and liver tissue should also be carefully assessed at this timepoint to assess for safety. Secondly, the two dosages tested changed multiple parameters at once, including ultrasound power and frequency, time of administration, and microbubble concentration. Further investigation of which parameters are most influential in the varying effects of AVUS and the point at which perfusion effects transition from an increase to disruption are necessary to achieve these effects in larger animals or humans. Lastly, the pulse sequences delivered can be optimized to enhance therapy and penetration of acoustic energy into the central part of tumor. Although the current therapeutic protocol involving intermittent insonations allows for damage to the outer vessels in the early treatment to allow increased penetration of US in the latter part of the treatment, a more elegant approach of interleaving flash replenishment sequences to the treatment to enhance ultrasound penetration may provide significant improvement in the treatment response and also reduce variability.

In conclusion, this study was able to translate AVUS into a realistic model of HCC, evaluating the feasibility of affecting authentic tumor vascular in situ. We found that AVUS induced significant vascular changes. A reduced dose was found to be ineffective at disrupting blood flow but could possibly have potential for enhancing blood flow. Further studies of the long-term effect and benefit of AVUS in HCC will inform its potential for further translation and implementation as a noninvasive locoregional therapy for HCC.
