# Supplementary material for: Microbubble-enhanced ultrasound for the antivascular treatment and monitoring of hepatocellular carcinoma
Source: Nanotheranostics. 2019 Oct 1;3(4):331–41. doi: 10.7150/ntno.39514 (PMC6821993; doi:10.7150/ntno.39514)
Supplement: Supplementary file 1 — Supplementary figures and tables. [file ntnov03p0331s1.pdf]

Supplementary Information

Table S1: Contrast enhanced ultrasound quantitative parameters before and after AVUS

	**Sham**			**Primary Dose AVUS**			**Reduced Dose AVUS**		
	Pre Mean(±SEM)	Post Mean(±SEM)	P-Value	Pre Mean(±SEM)	Post Mean(±SEM)	P-Value	Pre Mean(±SEM)	Post Mean(±SEM)	P-Value
**NLC Peak Enhancement**	3.9 (2.4)	3.1 (1.7)	*0.17*	5.3 (1.3)	2.8 (0.7)	*0.003*	4.7 (1.5)	6.9 (2.7)	*0.90*
**NLC Perfusion Index**	4.0 (2.7)	3.2 (1.7)	*0.20*	4.7 (1.2)	2.8 (0.7)	*0.007*	3.6 (1.0)	6.7 (2.7)	*0.93*
**NLC Time to Peak**	16.8 (2.8)	25.3 (7.3)	*0.07*	16.5 (1.8)	24.6 (3.0)	*0.009*	23.1 (2.6)	26.8 (4.9)	*0.26*
**PD Peak Perfused Area**	79.3 (9.9)	76.4 (9.1)	*0.16*	87.2 (3.6)	73.2 (6.0)	*0.014*	67.5 (9.3)	68.2 (8.6)	*0.54*
**PD Perfusion Area Index**	95.6 (15.9)	88.8 (16.7)	*0.26*	104.3 (7.5)	77.8 (8.2)	*0.009*	82.7 (14.4)	77.1 (15.9)	*0.23*
**PD Color Weighted Fractional Area Peak Enhancement**	40.6 (6.6)	37.4 (6.7)	*0.04*	48.0 (3.0)	35.5 (3.6)	*0.007*	37.7 (6.6)	33.6 (3.7)	*0.22*
**Delta Projection Area**	57.6 (10.0)	58.2 (10.2)	*0.63*	75.7 (4.2)	59.1 (6.4)	*0.002*	66.8 (8.2)	64.3 (7.8)	*0.29*
